# Evaluation of the Anatomical Cross-Sectional Area of Psoas Major Muscle Using an Ultrasound Imaging System Combined With an Inertial Measurement Unit: Improved Reliability in the US Using IMU-Based Positioning Techniques

**DOI:** 10.1155/2024/7774612

**Published:** 2024-10-29

**Authors:** Kazuhiro Ito, Etsuko Maeshima, Nobuyuki Arai, Koichi Saito, Hiroya Koshiba, Junko Maruyama, Keiji Asada, Takaaki Nakamata, Kazuki Yamaguchi, Yasuhiko Hatanaka

**Affiliations:** ^1^Department of Rehabilitation Physical Therapy Course, Faculty of Health Science, Suzuka University of Medical Science, 1001-1, Kishioka, Suzuka, Mie 510-0293, Japan; ^2^Graduate School of Sport and Exercise Sciences, Osaka University of Health and Sport Sciences, 1-1 Asadaidai, Kumatori, Sennan-Gun, Osaka 590-0496, Japan; ^3^Department of Radiological Technology, Faculty of Health Science, Suzuka University of Medical Science, 1001-1, Kishioka, Suzuka, Mie 510-0293, Japan; ^4^Department of Clinical Engineering, Faculty of Medical Engineering, Suzuka University of Medical Science, 1001-1, Kishioka, Suzuka, Mie 510-0293, Japan

**Keywords:** anatomical muscle cross-sectional area, B-mode ultrasonography, imaging method, inertial measurement unit, magnetic resonance imaging, muscle thickness

## Abstract

**Introduction:** Recently, ultrasound (US) imaging has been used to estimate the cross-sectional area of skeletal muscle, but the reliability is uncertain. To improve the reliability of the US, we investigated skeletal muscle thickness measurement using an inertial measurement unit (IMU) to determine the direction of US beam incidence based on posture angle information. In addition, we examined whether the anatomical cross-sectional area (ACSA) of muscle can be estimated from the muscle thickness measured using the US with the IMU.

**Methods:** In Experiment 1, two examiners measured the right psoas major at the fourth lumbar vertebra level in 10 university students using the US with and without an IMU. The intraclass correlation coefficient (ICC) was used to examine intra- and inter-rater variability. In Experiment 2, the two examiners measured the muscle thickness of the right psoas major in 31 male subjects using the US with an IMU. In addition, the ACSA of this muscle was measured using MRI. Pearson's correlation coefficient was used to examine the relationship between muscle thickness and ACSA, and a single regression analysis was performed.

**Results:** Both intrarater reliability ICC (1, 2) and inter-rater reliability ICC (2, 2) were higher when US was used with IMU compared to without IMU (Experiment 1). A significant positive correlation (*r* = 0.84, *p* < 0.01) was observed between muscle thickness and ACSA (Experiment 2). The regression equation was significant at *R*^2^ = 0.71 (*p* < 0.01).

**Conclusion:** Using an IMU during US measurement of the psoas major improves intra- and interexaminer reliability and can be used to estimate the ACSA of the muscle.

## 1. Introduction

Skeletal muscle declines in strength and mass with age. It has been reported that the muscle of the lower limb that exhibits the most significant decrease in muscle mass with age is the psoas major [1]. The psoas major is active during the latter half of the stance phase and the first half of the swing phase of gait and plays an essential role in increasing gait speed [2]. Moreover, the psoas major contributes to trunk stability in the antigravity posture [3, 4], making it the muscle that most reflects the decline in activity associated with aging [5]. The loss of muscle mass that occurs with aging is referred to as sarcopenia, a progressive and generalized loss of skeletal muscle mass and strength associated with an increased risk of reduced physical function and quality of life [6, 7]. The European Working Group on Sarcopenia in Older People (EWGSOP) suggests that the muscle mass of the lower back, including the psoas major, may be a valuable indicator of sarcopenia in the future [8]. Consequently, developing a practical methodology for quantifying psoas major muscle mass is a crucial area of research.

Various imaging methods are utilized to quantitate muscle mass, including magnetic resonance imaging (MRI), computerized tomography (CT), and ultrasound (US) imaging [9], as well as dual-energy X-ray absorptiometry (DXA) [10] and bioelectrical impedance analysis (BIA) [11]. MRI, CT, and DXA are expensive and require a large measurement environment, while CT and DXA have the disadvantage of radiation exposure. US is inexpensive, portable, and noninvasive compared to MRI and CT. It is a handy tool for measuring muscle mass because it allows real-time, repeated observation of the morphology of individual skeletal muscles.

When using the US, measuring the anatomical cross-sectional area (ACSA) of a muscle is challenging unless the muscle is small enough for the US beam from the probe to capture the entire muscle. In addition, a significant drawback is that the examiner's proficiency has a significant effect on the reliability of muscle thickness measurements [12]. In recent years, techniques have been developed to create three-dimensional reconstructions of biological tissues and to assess the cross-sectional area of these tissues using multiple US images [13–15]. However, challenges remain, including technologies for maintaining consistent probe positioning, angle, and pressure, as well as for removing noise to construct accurate 3D models. Therefore, we consider that at the current stage, the most practical method for evaluating muscle mass using the US is to estimate ACSA based on the results of precise muscle thickness measurements.

Measurement errors in US muscle thickness measurement can be caused by the selection of an inappropriate probe, the position and orientation of the probe relative to the tissue to be visualized, and changes in pressure when the probe is in contact with the skin [16]. Currently, averaging multiple measurements by multiple skilled examiners is used to ensure the reliability of US measurements [17, 18]. Therefore, a method for measuring muscle thickness using the US with objective support to minimize measurement error is needed.

The inertial navigation system (INS) is the technology used to obtain information on the position and orientation of objects in inertial space. The INS determines the position and orientation of a sensor using measurement information from an inertial measurement unit (IMU). The main IMU consists of a 3-axis accelerometer, a 3-axis angular rate sensor, and a 3-axis magnetometer module.

In addition, a Bluetooth communication module enables the real-time acquisition of the 3-dimensional attitude angle of the sensor without any limitation in the measurement space. In recent years, the development of microelectromechanical systems' (MEMSs) technology has led to the miniaturization, cost reduction, and performance improvement of sensors, which are now widely used in kinematic studies of living organisms [19, 20]. Furthermore, the advancement of sensor fusion technology has made it possible to compensate for various measurement errors and accurately determine position and orientation.

This study aimed to verify the hypothesis that employing INS technology to determine the posture angle of the probe used in US measurements facilitates precise measurements with minimal measurement error. The study was initiated by verifying whether intra- and interexaminer reliability in measuring muscle thickness of the psoas major was improved by using the posture angle information obtained by the IMU to determine the direction of incidence of the ultrasonic beam. Furthermore, we sought to ascertain whether estimating the ACSA of the psoas major is feasible from the muscle thickness values obtained by the combined IMU measurements.

## 2. Methods

### 2.1. Participants

Subjects were university students who indicated their willingness to cooperate in the study in response to a leaflet outlining the study's purpose and methods and a verbal invitation to participate. Exclusion criteria were a history of osteoarticular or neurological disease or surgery.

This study was conducted according to the Declaration of Helsinki after fully explaining the study's purpose and experimental methods to the subjects in advance, obtaining their written consent, and receiving approval from the Ethics Committee at Suzuka University of Medical Science (no. 563).

### 2.2. Experiment 1: Comparison of Intra- and Interexaminer Reliability in Measuring Psoas Major Muscle Thickness Using the US With and Without IMU

A total of 10 male subjects participated in the study. The participants had a mean (mean ± standard deviation) age of 20.1 ± 0.3 years, a mean height of 173.4 ± 5.4 cm, a mean weight of 63.1 ± 9.5 kg, and a mean BMI of 20.9 ± 2.5 kg/m^2^.

#### 2.2.1. Measurement of Psoas Major Muscle Thickness

**Figure 1:**
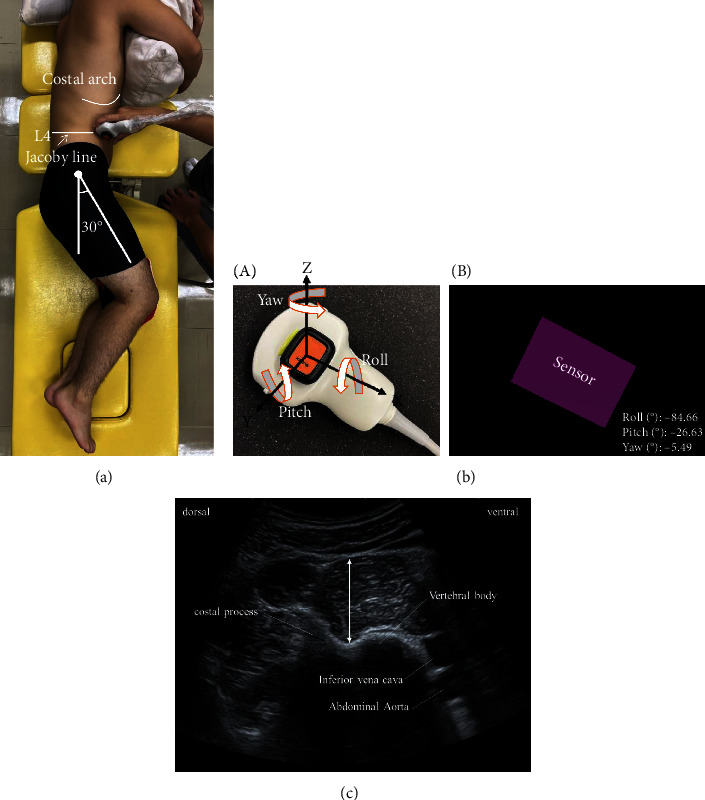
Experimental setup for US measurement. (a) The subject was positioned in a left lateral decubitus position and both hip joints flexed at approximately 30°. (b) (A) The position where the IMU is attached on the probe. (B) The three-dimensional posture angles (roll, pitch, and yaw) of the probe (purple square), as measured by the IMU, are displayed on the PC screen. (c) Ultrasound image of the psoas major. Arrow: muscle thickness of the psoas major.

The US imaging system Xario100 (Canon, Tokyo, Japan) was used. The imaging site was positioned at the level of the fourth lumbar vertebra, which is considered the position with the greatest ACSA of the psoas major [21]. In a previous study measuring psoas major muscle thickness, the measurement posture was prone, and imaging was performed from the lumbar back [22]. We attempted to use the same method but found that capturing the high-luminance region just below the fascia was difficult. In addition, we assumed that elderly patients could not be placed in the prone position for measurement. Thus, we attempted to capture images from the ventral side of the subject in a lateral decubitus position, with the imaging side up. Compared to imaging from the lumbar back, the distance from the probe to the psoas major is greater, and it is difficult to capture the fascial boundary clearly due to beam attenuation at high frequencies. Therefore, a 3.5 MHz convex probe was used. The right side was designated for measurements, with the subject positioned in a left lateral decubitus position and both hip joints flexed at approximately 30°. The probe was applied from the lateral abdomen at the level of the fourth lumbar vertebra, using the Jacoby line as a guide. Short-axis images were taken at the end of the expiratory phase to minimize the influence of the respiratory musculature. The psoas major, abdominal aorta, inferior vena cava, costal process, and vertebral body were successfully visualized (Figure 1(a)).

Two examiners, Examiner A (an experienced US operator) and Examiner B (an inexperienced US operator), conducted the measurements. Examiner A had more than 3 years of experience in performing US imaging of the psoas major muscle, while Examiner B had only about 1 hour of experience in operating the US probe. Each examiner performed muscle thickness measurements twice: once with the IMU installed in the probe and once without the IMU. The Xsens DOT IMU (Movella Technologies, Enschede, Netherlands) was used. For both examiners, whether measurements were taken under the condition with or without the IMU was randomly determined for each subject. Measurements were taken on the same day for each examiner. When an IMU was used, the 3D posture angle of the probe was measured at the time of the first imaging, and the second imaging was performed according to the posture angle of the probe at the time of the first imaging (Figure 1(b)). For muscle thickness measurement, ImageJ (National Institutes of Health, Bethesda, Maryland, USA) was used to measure the distance between the nadirs of the hyperintensities (fascia) (Figure 1(c)).

#### 2.2.2. Statistical Analysis

Statistical analysis was performed using SPSS ver. 28 (IBM, Japan). First, after confirming the normality of the data with the Shapiro–Wilk test, the intraclass correlation coefficient (ICC) was used to examine the intra- and inter-rater reliability of the muscle thickness measurements with and without the IMU. Intrarater reliability refers to the degree of agreement when a single rater performs multiple measurements on multiple subjects. For intrarater reliability, ICC (1, 1) was calculated for the first and second measurements of Examiners A and B, and ICC (1, 2) was calculated for the average of the two measurements of Examiners A and B, respectively. Inter-rater reliability refers to the degree of agreement when multiple raters conduct multiple measurements on multiple subjects. For inter-rater reliability, ICC (2, 2) was calculated for the mean of the two measurements of Examiners A and B. Based on the criteria of Landis and Koch [23], ICC values of 0.41–0.60 were considered moderate, 0.61–0.80 were considered substantial, and 0.81–1.00 were considered almost perfect. Absolute reliability was also assessed.

Bland–Altman analysis was used to check for the presence of additive and proportional errors and confirm absolute reliability. The standard error of measurement (SEM), an index of absolute reliability, was also calculated. The minimal detectable change at 95% confidence (MDC_95_) was calculated by multiplying SEM by 1.96.

### 2.3. Experiment 2: The Relationship Between Psoas Major Muscle Thickness and ACSA

A total of 31 male subjects participated in the study. The participants had a mean (mean ± standard deviation) age of 21.4 ± 0.8 years, a mean height of 171.6 ± 6.5 cm, a mean weight of 62.8 ± 9.5 kg, and a mean BMI of 21.3 ± 2.5 kg/m^2^.

#### 2.3.1. Measurement of Psoas Major Muscle Thickness

Two examiners (Examiners C and D, inexperienced US operators) performed the measurements using the same method as in Experiment 1 with the IMU. The two examiners were inexperienced in US measurement and practiced the probe operation only about 1 hour before the start of the experiment.

#### 2.3.2. Measurement of Psoas Major Muscle ACSA

The position of the subject during imaging was maintained in the same lateral decubitus position, with both hip joints flexed at approximately 30°, as during the US measurements. All examinations were performed on a 1.5T MRI system (Echelon Vega, FUJIFILM Healthcare Corporation, Tokyo, Japan) with an 8-channel body matrix coil. The fast spin echo technique is the method used to acquire the muscle's morphological features, which had the following parameters: repetition time (TR), 450 ms; echo time (TE), 10 ms; flip angle, 90°; slice thickness, 7 mm; matrix, 352 × 352; field of view, 352 mm (in-plane resolution, 1.00 × 1.00 mm); echo train length, 3; number of signal averages (NSA), 2; receiver bandwidth, 68.5 kHz; and acquisition time, 1 min 20 s. To acquire the apparent diffusion coefficient (ADC), the diffusion-weighted images were as follows: TR, 4000 ms; TE, 65 ms; flip angle, 90°; slice thickness, 7 mm; matrix, 192 × 192; field of view, 352 mm (in-plane resolution, 1.83 × 1.83 mm); parallel imaging factor, 2.0; NSA, 6; receiver bandwidth, 384.7 kHz; *b*-values, 0 and 1000 s/mm^2^; and acquisition time, 1 min 40 s. The measurement site for the psoas major muscle was set at the lower end of the fourth lumbar vertebra. ACSA of the right psoas major was determined by tracing its perimeter on MR images.

#### 2.3.3. Statistical Analysis

Statistical analysis was performed using SPSS ver. 28 (IBM, Japan). After confirming the normality of the data with the Shapiro–Wilk test, ICCs were calculated to assess intrarater reliability (ICC [1, 1], ICC [1, 2]) and inter-rater reliability (ICC [2, 2]). In addition, absolute reliability was evaluated using Bland–Altman analysis to examine the presence of fixed bias and proportional bias, and the SEM was calculated. Furthermore, the MDC_95_ was determined.

To assess the validity of muscle thickness measurements by the US, Pearson's correlation coefficient was used to examine their relationship with ACSA measured by MRI. Moreover, to estimate ACSA from muscle thickness measurements, a simple linear regression analysis was conducted with ACSA as the dependent variable and muscle thickness as the independent variable. A value of *p* < 0.05 was regarded as statistically significant.

## 3. Results

### 3.1. Experiment 1

All measurements were confirmed to be normally distributed using the Shapiro–Wilk test. The results of the psoas major muscle thickness measurements of Examiners A and B are presented in Table 1.

**Figure 2:**
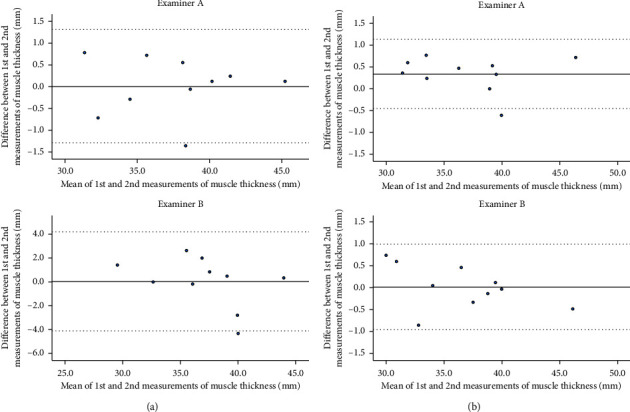
Bland–Altman plot showing the difference between the first and second measurements of psoas major muscle thickness in relation to the mean of the two measurements taken by one examiner on a subject. The solid line represents the mean difference between the first and second measurements. The dashed line represents the upper and lower limits of agreement. (a) Without IMU. (b) With IMU.

The intrarater reliability of ICC (1, 1) ranged from 0.989 to 0.994 and ICC (1, 2) from 0.994 to 0.997 for Examiner A, an experienced examiner, with no notable difference observed between measurements taken with and without the IMU. In contrast, ICC (1, 1) and ICC (1, 2) improved for Examiner B, an inexperienced examiner, from 0.883 to 0.995 and from 0.938 to 0.998, respectively. Bland–Altman analysis showed no fixed bias or proportional bias for either Examiner A or Examiner B (Figure 2). For Examiner A, SEM decreased from 0.47 to 0.29 and MDC_95_ decreased from 1.30 to 0.79 mm. For Examiner B, SEM decreased from 1.51 to 0.35 and MDC_95_ was markedly reduced from 4.17 to 0.97 mm (Table 2).

**Figure 3:**
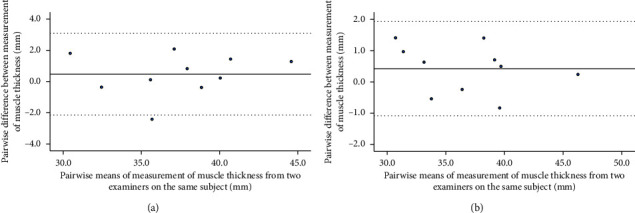
Bland–Altman plot showing the difference between the mean values of two measurements of psoas major muscle thickness taken by two examiners, in relation to the average of the measurement from the same two examiners on a single subject. The solid line represents the mean difference between the first and second measurements. The dashed lines represent the upper and lower limits of agreement. (a) Without IMU. (b) With IMU.

Inter-rater reliability improved from 0.973 to 0.988 for ICC (2, 2) without and with IMU. Bland-Altman analysis showed no fixed bias or proportional bias in either case (Figure 3), with SEM decreasing from 0.94 to 0.54 and MDC_95_ decreasing from 2.60 to 1.50 mm (Table 3).

### 3.2. Experiment 2

All measurements were confirmed to be normally distributed using the Shapiro–Wilk test. The results of the psoas major muscle thickness measurements for Examiners C and D are presented in Table 4.

**Figure 4:**
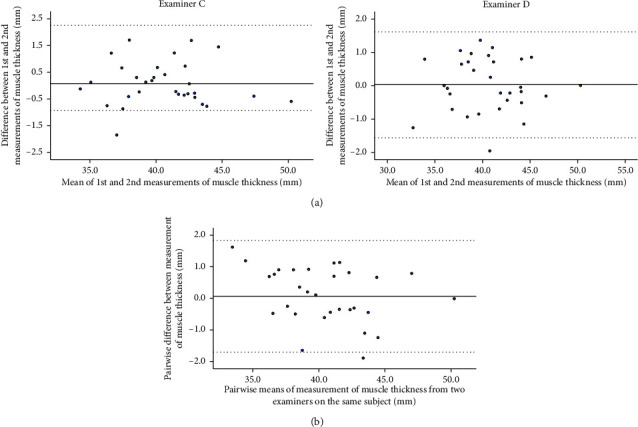
(a) Bland–Altman plot showing the difference between the first and second measurements of psoas major muscle thickness in relation to the mean of the two measurements taken by one examiner on a subject. The solid line represents the mean difference between the first and second measurements. The dashed line represents the upper and lower limits of agreement. (b) Bland–Altman plot showing the difference between the mean values of two measurements of psoas major muscle thickness taken by two examiners, in relation to the average of the measurement from the same two examiners on a single subject. The solid line represents the mean difference between the first and second measurements. The dashed lines represent the upper and lower limits of agreement.

The results of intrarater reliability were as follows: ICC (1, 1) was 0.974 for Examiner C and 0.979 for Examiner D, and ICC (1, 2) was 0.987 for Examiner C and 0.989 for Examiner D. Bland–Altman analysis showed no fixed bias or proportional bias (Figure 4(a)), with SEM at 0.57 mm for Examiner C and 0.58 mm for Examiner D, and MDC_95_ at 1.59 mm for both Examiners C and D (Table 5).

The inter-rater reliability result in ICC (2, 2) was 0.985. Bland–Altman analysis showed no fixed bias or proportional bias (Figure 4(b)), with SEM at 0.64 mm and MDC_95_ at 1.76 mm (Table 6).

**Figure 5:**
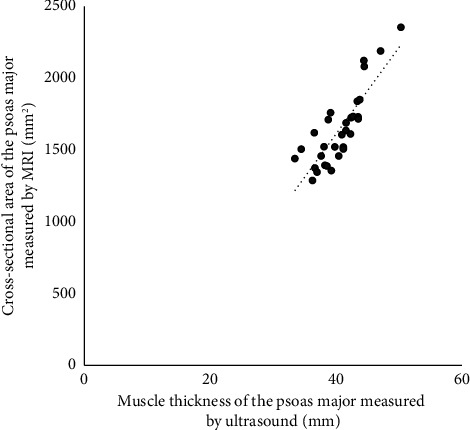
The relationship between muscle cross-sectional area and muscle thickness of the psoas major.

Pearson's correlation analysis of muscle thickness and ACSA of the psoas major revealed a significant correlation (*r* = 0.84, *p* < 0.01). Single regression analysis showed that the obtained regression equation was ACSA = 60.2 × muscle thickness − 798.4, and the coefficient of determination *r*^2^ was significant at 0.71 (*p* < 0.01) (Figure 5).

## 4. Discussion

In Experiment 1, results confirmed that incorporating an IMU in the US measurement of the psoas major muscle improved both intrarater and inter-rater reliability. This improvement was particularly notable for the intrarater reliability of the inexperienced examiner, suggesting that the use of an IMU can mitigate measurement errors caused by inconsistent probe posture angles, regardless of the examiner's experience level. Previous studies have reported high intrarater and inter-rater reliability for US muscle thickness measurements of the limbs and trunk muscles [24–26]. In Experiment 2, even with measurements conducted by two inexperienced examiners, intrarater and inter-rater reliability was comparable to these previous studies. In addition, with an MDC_95_ of 1.76 mm, when using the average of two measurements from each examiner, it was determined that a change of 1.76 mm or more in psoas major muscle thickness measured by this method could be considered significant. Furthermore, Bland–Altman analysis revealed no systematic bias for both intrarater and inter-rater reliability, indicating that this method, which uses the average of two measurements by two examiners, can minimize the risk of systematic bias and, thus, is suitable for clinical application.

The necessity of imaging the entire muscle when measuring ACSA by the US renders accurate measurement challenging, especially for relatively superficial, small, and thin muscles. Therefore, it is necessary to verify whether ACSA can be estimated from muscle thickness measurements obtained via the US to effectively capture muscle volume changes. Previous studies have found that ACSA of various muscles, such as the quadriceps muscle (*r* = 0.91, *n* = 52) [27], hip adductor muscle (*r* = 0.92, *n* = 20) [28], tibialis anterior muscle (*r* = 0.90, *n* = 17) [29], gastrocnemius muscle (*r* = 0.91, *n* = 6) [30], pectoralis major muscle (*r* = 0.92, *n* = 20) [31], forearm muscle group (*r* = 0.94, *n* = 10), and radial forearm muscle group (*r* = 0.88, *n* = 10) [32], could be estimated from muscle thickness measurements. For the psoas major, the subject of this study, Takai et al. [22] reported a correlation coefficient of *r* = 0.95 (*n* = 11), while Ikezoe et al. [1] reported *r* = 0.97 (*n* = 16). In our study, despite the examiners being inexperienced, a high correlation of *r* = 0.84 (*n* = 31) was observed, which is comparable to previous studies on the psoas major and other muscles. This result demonstrates that even inexperienced examiners can accurately estimate ACSA, highlighting the value of our findings. US measurements using this method are low-cost and noninvasive and provide reliable estimates of psoas major muscle mass changes, thus, making them suitable for regular assessments. Consequently, this method has the potential for early detection and prevention of sarcopenia in older people. In addition, it allows for the easy evaluation of muscle mass changes over time due to psoas major strengthening exercises, offering an objective basis for continuing or modifying training programs.

In addition to muscle thickness measurements, muscle mass evaluation by the US includes the estimation of physiological cross-sectional area (PCSA), ACSA, and muscle volume from pennation angle and fascicle length [33]. Muscle quality assessment also includes echo intensity measurements that reflect the amount of intramuscular fat and noncontractile elements of connective tissue within the muscle [34]. In these measurements, changes in the direction of incidence of the US beam greatly affect the measurement results. Therefore, the present method, which can determine the posture angle of the probe in combination with the IMU, can be applied to minimize measurement errors in these measurement methods as well. However, the subjects of this study were male university students, and it is necessary to confirm the reliability and validity of the technique for use in women and the elderly in the future.

## 5. Conclusion

The use of IMU with US measurement of psoas major muscle thickness improved intra- and inter-rater reliability, even for inexperienced examiners. In addition, since psoas major muscle thickness measured by this method can be used to estimate the ACSA, it is expected to be applied in the detection and prevention of sarcopenia, as well as in the assessment of training effectiveness.

## Figures and Tables

**Table 1:** Muscle thickness of psoas major.

Examiner	Without IMU		With IMU	
	First (mm)	Second (mm)	First (mm)	Second (mm)
A (experienced)	37.58 ± 4.04	37.57 ± 4.04	37.21 ± 4.39	36.87 ± 4.44
B (inexperienced)	37.14 ± 3.55	37.09 ± 4.36	36.63 ± 4.51	36.62 ± 4.73

*Note:* Data are expressed as mean ± SD.

Abbreviation: IMU, inertial measurement unit.

**Table 2:** Intrarater reliability of psoas major muscle thickness measurements without and with IMU.

IMU	Examiner	ICC (1, 1)	ICC (1, 2)	Bland–Altman analysis				SEM	MDC_95_
				Fixed bias		Proportional bias			
		95% CI		95% CI	Y/N	Regression coefficient	Y/N		
Without	A (experienced)	0.989	0.994	−0.47∼0.48	N	0.007	N	0.47	1.30
		0.959∼0.997	0.979∼0.999						
	B (inexperienced)	0.883	0.938	−1.47∼1.57	N	0.411	N	1.51	4.17
		0.619∼0.969	0.765∼0.984						

With	A (experienced)	0.994	0.997	−0.25∼0.53	N	0.16	N	0.29	0.79
		0.977∼0.998	0.988∼0.999						
	B (inexperienced)	0.995	0.998	−0.34∼0.37	N	0.47	N	0.35	0.97
		0.982∼0.999	0.991∼0.999						

Abbreviations: 95% CI, 95% confidence interval; ICC, intraclass correlation coefficient; IMU, inertial measurement unit; MDC_95_, minimal detectable change at 95%; SEM, standard error of measurement.

**Table 3:** Inter-rater reliability of psoas major muscle thickness measurements without and with IMU.

IMU	ICC (2, 2)	Bland–Altman analysis				SEM	MDC_95_
		Fixed bias		Proportional bias			
	95% CI	95% CI	Y/N	Regression coefficient	Y/N		
Without	0.973	−0.48∼1.42	N	0.15	N	0.94	2.60
	0.898∼0.993						

With	0.988	−0.13∼0.97	N	0.29	N	0.54	1.50
	0.968∼0.997						

Abbreviations: 95% CI, 95% confidence interval; ICC, intraclass correlation coefficient; IMU, inertial measurement unit; MDC_95_, minimal detectable change at 95%; SEM, standard error of measurement.

**Table 4:** Muscle thickness of psoas major.

Examiner	First (mm)	Second (mm)
C	40.65 ± 3.47	40.59 ± 3.50
D	40.57 ± 3.78	40.54 ± 3.80

*Note:* Data are mean ± SD.

**Table 5:** Intrarater reliability in the measurement of psoas major muscle thickness.

Examiner	ICC (1, 1)	ICC (1, 2)	Bland–Altman analysis				SEM	MDC_95_
			Fixed bias		Proportional bias			
	95% CI		95% CI	Y/N	Regression coefficient	Y/N		
C (inexperienced)	0.974	0.987	−0.23∼0.36	N	0.04	N	0.58	1.59
	0.948∼0.987	0.973∼0.994						

D (inexperienced)	0.979	0.989	−0.27∼0.32	N	0.02	N	0.57	1.59
	0.956∼0.990	0.978∼0.995						

Abbreviations: 95% CI, 95% confidence interval; ICC, intraclass correlation coefficients; IMU, inertial measurement unit; MDC_95_, minimal detectable change 95; SEM, standard error of measurement.

**Table 6:** Inter-rater reliability in the measurement of psoas major muscle thickness.

ICC (2, 2)	Bland–Altman analysis				SEM	MDC_95_
	Fixed bias		Proportional bias			
95% CI	95% CI	Y/N	Regression coefficient	Y/N		
0.985	−0.26∼0.40	N	0.35	N	0.64	1.76
0.969–0.993						

Abbreviations: 95% CI, 95% confidence interval; ICC, intraclass correlation coefficient; MDC_95_, minimal detectable change at 95%; SEM, standard error of measurement.

## Data Availability

Measurement data are available as supporting information.

## Supporting Information

Supporting Table 1 presents the raw data from Experiment 1. Supporting Table 2 presents the raw data from Experiment 2.
