# Supplementary material for: Evaluation of the Anatomical Cross-Sectional Area of Psoas Major Muscle Using an Ultrasound Imaging System Combined With an Inertial Measurement Unit: Improved Reliability in the US Using IMU-Based Positioning Techniques
Source: Transl Sports Med. 2024 Oct 29;2024:7774612. doi: 10.1155/2024/7774612 (PMC11537743; doi:10.1155/2024/7774612)
Supplement: Supporting Information — Additional supporting information can be found online in the Supporting Information section. [file 7774612.f1.zip › Supplementary Table 1.pdf]

subject characteristics						Muscle thickness of psoas major (mm)							
						Without IMU				With IMU			
						Examiner A		Examiner B		Examiner A		Examiner B	
ID	Sex	age (years)	Height (m)	Weight (kg)	BMI (kg/m^2)	first	second	first	second	first	second	first	second
1	male	20	1.79	82	25.6	40.23	40.11	38.54	41.33	39.65	40.26	39.52	39.40
2	male	20	1.72	65	22.0	41.55	41.31	37.82	42.16	38.93	38.93	37.37	37.70
3	male	20	1.74	75	24.8	37.67	39.03	37.95	37.10	39.67	39.34	38.74	38.87
4	male	21	1.63	53	19.9	31.75	30.97	30.27	28.84	31.59	31.23	30.38	29.64
5	male	20	1.68	55	19.5	36.01	35.29	36.85	34.21	36.50	36.03	36.74	36.28
6	male	20	1.8	60	18.5	34.34	34.63	37.89	35.87	33.62	33.38	34.06	34.01
7	male	20	1.74	55	18.2	31.92	32.64	32.64	32.64	32.16	31.56	31.20	30.60
8	male	20	1.76	68	22.0	38.42	37.87	35.98	36.15	33.84	33.07	32.40	33.25
9	male	20	1.8	66	20.4	45.29	45.17	44.13	43.78	46.73	46.01	45.90	46.38
10	male	20	1.68	52	18.4	38.63	38.69	39.28	38.78	39.44	38.91	39.99	40.02

IMU: inertial measurement unit
