# Supplementary material for: Evaluation of the Anatomical Cross-Sectional Area of Psoas Major Muscle Using an Ultrasound Imaging System Combined With an Inertial Measurement Unit: Improved Reliability in the US Using IMU-Based Positioning Techniques
Source: Transl Sports Med. 2024 Oct 29;2024:7774612. doi: 10.1155/2024/7774612 (PMC11537743; doi:10.1155/2024/7774612)
Supplement: Supporting Information — Additional supporting information can be found online in the Supporting Information section. [file 7774612.f1.zip › Supplementary Table 2.pdf]

| subject characteristics | | | | | | Muscle thickness of psoas major (mm) | | | |
|---|---|---|---|---|---|---|---|---|---|
| | | | | | | Examiner C | | Examiner D | |
| ID | Sex | age (years) | Height (m) | Weight (kg) | BMI (kg/m2) | first | second | first | second |
| 1 | male | 22 | 1.64 | 49 | 18.2 | 35.91 | 36.67 | 36.42 | 37.13 |
| 2 | male | 22 | 1.68 | 73 | 25.9 | 39.29 | 39.16 | 39.26 | 38.80 |
| 3 | male | 21 | 1.73 | 58 | 19.4 | 41.55 | 41.88 | 41.05 | 40.15 |
| 4 | male | 21 | 1.69 | 62 | 21.7 | 42.57 | 41.85 | 42.35 | 42.78 |
| 5 | male | 22 | 1.77 | 85 | 27.1 | 49.99 | 50.59 | 50.30 | 50.29 |
| 6 | male | 22 | 1.67 | 64 | 22.9 | 35.11 | 35.00 | 34.26 | 33.47 |
| 7 | male | 21 | 1.71 | 59 | 20.2 | 43.54 | 41.85 | 41.77 | 41.99 |
| 8 | male | 21 | 1.8 | 58 | 17.9 | 42.56 | 42.50 | 42.73 | 42.95 |
| 9 | male | 22 | 1.8 | 72 | 22.2 | 47.25 | 47.65 | 46.51 | 46.82 |
| 10 | male | 22 | 1.71 | 57 | 19.5 | 38.68 | 38.38 | 38.16 | 37.11 |
| 11 | male | 22 | 1.67 | 65 | 23.3 | 37.71 | 38.13 | 39.15 | 40.00 |
| 12 | male | 22 | 1.74 | 63 | 20.8 | 43.48 | 44.26 | 45.53 | 44.68 |
| 13 | male | 22 | 1.62 | 50 | 19.1 | 36.09 | 37.95 | 36.22 | 36.30 |
| 14 | male | 22 | 1.65 | 55 | 20.2 | 39.99 | 39.70 | 40.41 | 39.05 |
| 15 | male | 21 | 1.81 | 73 | 22.3 | 38.82 | 37.12 | 38.82 | 38.11 |
| 16 | male | 21 | 1.81 | 63 | 19.2 | 42.00 | 40.78 | 41.40 | 42.09 |
| 17 | male | 22 | 1.6 | 49 | 19.1 | 41.40 | 41.63 | 40.93 | 40.68 |
| 18 | male | 22 | 1.64 | 70 | 26.0 | 34.19 | 34.32 | 32.00 | 33.26 |
| 19 | male | 21 | 1.68 | 55 | 19.5 | 37.21 | 36.00 | 35.91 | 35.91 |
| 20 | male | 23 | 1.64 | 60 | 22.3 | 38.60 | 38.84 | 37.91 | 38.84 |
| 21 | male | 23 | 1.78 | 58 | 18.3 | 40.88 | 40.47 | 41.47 | 40.76 |
| 22 | male | 20 | 1.73 | 65 | 21.7 | 41.96 | 42.33 | 41.59 | 40.45 |
| 23 | male | 20 | 1.77 | 73 | 23.3 | 42.79 | 43.09 | 43.95 | 44.13 |
| 24 | male | 21 | 1.6 | 43 | 16.8 | 37.05 | 37.93 | 38.06 | 37.42 |
| 25 | male | 22 | 1.72 | 64 | 21.6 | 40.45 | 39.78 | 39.76 | 41.71 |
| 26 | male | 21 | 1.71 | 53 | 18.1 | 37.74 | 37.08 | 36.39 | 36.64 |
| 27 | male | 21 | 1.85 | 82 | 24.0 | 42.73 | 43.18 | 43.81 | 44.32 |
| 28 | male | 20 | 1.73 | 66 | 22.1 | 42.27 | 42.58 | 43.74 | 44.89 |
| 29 | male | 21 | 1.7 | 64 | 22.1 | 43.18 | 43.89 | 43.95 | 44.00 |
| 30 | male | 21 | 1.78 | 76 | 24.0 | 45.47 | 44.02 | 44.47 | 43.68 |
| 31 | male | 20 | 1.76 | 62 | 20.0 | 39.78 | 39.60 | 39.25 | 38.29 |

ACSA: anatomical cross-sectional area

| ACSA of psoas major ($mm^2$) |
|---:|
| 1619.5 |
| 1759.4 |
| 1520.4 |
| 1723.9 |
| 2353.3 |
| 1504.8 |
| 1611.3 |
| 1733 |
| 2188.8 |
| 1522.3 |
| 1710.6 |
| 2080.5 |
| 1374.2 |
| 1521.5 |
| 1393.6 |
| 1635.1 |
| 1506.2 |
| 1439.3 |
| 1287.3 |
| 1388.1 |
| 1605.4 |
| 1688.5 |
| 1730.7 |
| 1457.3 |
| 1457.6 |
| 1345.1 |
| 1715.6 |
| 1838.9 |
| 1850.1 |
| 2121.9 |
| 1356.5 |
